# Engineering Polyampholytes for Energy Storage Devices: Conductivity, Selectivity, and Durability

**DOI:** 10.3390/polym18010018

**Published:** 2025-12-21

**Authors:** Madina Mussalimova, Nargiz Gizatullina, Gaukhargul Yelemessova, Anel Taubatyrova, Zhanserik Shynykul, Gaukhar Toleutay

**Affiliations:** 1 Department of Chemical and Biochemical Engineering, Geology and Oil-Gas Business Institute Named After K. Turyssov, Satbayev University, Almaty 050043, Kazakhstan; mussalimova.m@stud.satbayev.university (M.M.); gizatullinanargiz.nz@gmail.com (N.G.); gauhargul1997@gmail.com (G.Y.); 0ani.lani9@gmail.com (A.T.); 2 Research Institute of Advanced Materials, Almaty 040000, Kazakhstan; 3 Department of Pharmaceutical Sciences, College of Pharmacy, The University of Tennessee Health Science Center, Memphis, TN 38163, USA; 4 Department of Chemistry, University of Tennessee, Knoxville, TN 37996, USA

**Keywords:** polyampholytes, polyzwitterions, gel polymer electrolytes, lithium metal batteries, zinc-ion batteries, supercapacitors, interfacial engineering

## Abstract

Polyampholytes combine cationic and anionic groups in one macromolecular platform and are emerging as versatile components for energy storage and conversion. This review synthesizes how their charge balance, hydration, and architecture can be engineered to address ionic transport, interfacial stability, and safety across batteries, supercapacitors, solar cells, and fuel cells. We classify annealed, quenched, and zwitterionic systems, outline molecular design strategies that tune charge ratio, distribution, and crosslinking, and compare device roles as gel or solid electrolytes, eutectogels, ionogels, binders, separator coatings, and interlayers. Comparative tables summarize ionic conductivity, cation transference number, electrochemical window, mechanical robustness, and temperature tolerance. Across Li and Zn batteries, polyampholytes promote ion dissociation, homogenize interfacial fields, suppress dendrites, and stabilize interphases. In supercapacitors, antifreeze hydrogels and poly(ionic liquid) networks maintain conductivity and elasticity under strain and at subzero temperature. In solar cells, zwitterionic interlayers improve work function alignment and charge extraction, while ordered networks in fuel cell membranes enable selective ion transport with reduced crossover. Design rules emerge that couple charge neutrality with controlled hydration and dynamic crosslinking to balance conductivity and mechanics. Key gaps include brittleness, ion pairing with multivalent salts, and scale-up. Opportunities include soft segment copolymerization, ionic liquid and DES plasticization, side-chain engineering, and operando studies to guide translation.

## 1. Introduction

**Figure 1:**
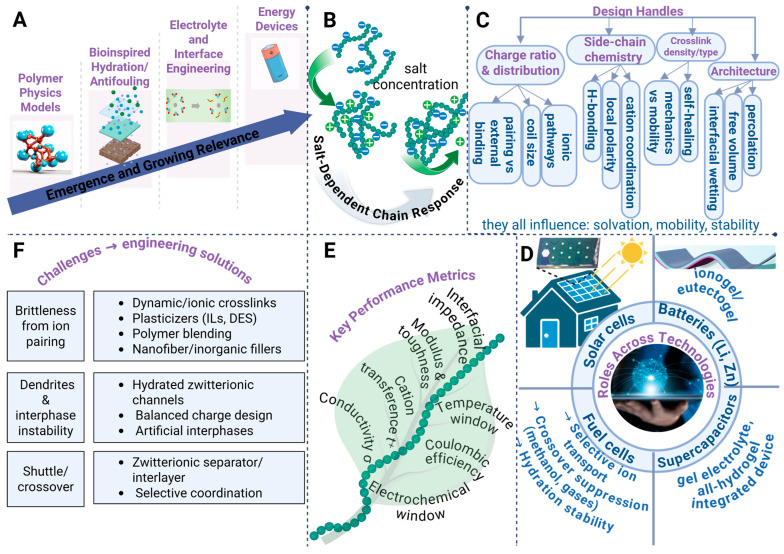
Design principles, structure–property relationships, and energy-device applications of polyampholytes: (**A**) Polyampholytes have evolved from classical charge-compensation concepts in polymer physics toward bioinspired, highly hydrated materials with increasing relevance for electrolyte and interface engineering in energy devices, as indicated by dashed vertical boundaries separating conceptual stages and a diagonal arrow denoting growing technological impact. (**B**) Salt-dependent chain responses distinguish classical polyelectrolytes from zwitterionic polyampholytes, where reversible expansion and contraction are driven by ionic strength (curved arrows), oppositely charged repeat units are shown as blue and green spheres, and light blue halos represent strongly bound hydration shells characteristic of zwitterions. (**C**) Ion transport and mechanical behavior are governed by molecular design handles including charge ratio and spatial distribution, side-chain chemistry (hydrogen bonding, local polarity, and cation coordination), crosslink density and type, and overall polymer architecture, which collectively regulate solvation, ionic mobility, and stability. (**D**) Key bottlenecks such as brittleness caused by ion pairing, interphase instability and dendrite formation, and shuttle or crossover effects are addressed through dynamic or ionic crosslinks, plasticization using ionic liquids or deep eutectic solvents, polymer blending, zwitterionic ion-conducting channels, selective coordination, and artificial interphases. (**E**) Electrochemical performance is summarized as a multidimensional property space comprising ionic conductivity, cation transference number, electrochemical stability window, interfacial impedance, Coulombic efficiency, mechanical robustness, and operational temperature range, emphasizing interdependent trade-offs among these metrics. (**F**) Across energy technologies including Li- and Zn-based batteries, supercapacitors, fuel cells, and solar cells, polyampholytes function as electrolytes, gels, ionogels, eutectogels, binders, separators, and interfacial layers, with arrows indicating ion transport, stress dissipation, and interfacial stabilization and color coding distinguishing device classes and functional roles.

Polyampholytes are classified as polymers that encompass both cationic and anionic moieties, thereby engendering coupled intra- and intermolecular electrostatic interactions that dictate solubility, hydration dynamics, chain conformation, and ionic transport in soft materials and electrolytic systems [1,2,3]. Historically, they emerged in polymer physics as model systems for charge compensation and coil–globule transitions, yet remained niche for several decades owing to synthetic constraints and limited application pull (Figure 1A) [4,5,6]. Interest in polyampholytes has proliferated as bioinspired design paradigms have elucidated the significance of tightly bound hydration shells and antifouling surface characteristics, thereby positioning polyampholytes as inherently water-compatible, salt-tolerant, and interface-friendly matrices [7,8,9]. In the preceding decade, there has been a consistent increase in scholarly publications and patents that reference zwitterionic and associated polyampholyte chemistries across the disciplines of materials science, and electrochemistry, indicating a paradigm shift from merely descriptive investigations toward the engineering of transport phenomena and interphase control [10,11]. These evolving trends are now converging within the realm of energy technologies, where hydrated domains, internal charge compensation, and selective ion coordination are deemed advantageous, thereby prompting a systematic evaluation of the design and implementation of polyampholytes across various applications, including batteries, supercapacitors, fuel cells, and solar energy systems.

The behavior of polyampholytes is dictated by a delicate equilibrium between the electrostatic attraction of oppositely charged entities and the screening effects induced by the incorporation of salts, which collectively influence the dimensions of the coils, their hydration state, and the mobility of ions [12]. Single-charge polyelectrolytes contract when added salt screens intrachain repulsion, whereas many zwitterions expand as salt weakens internal ion pairs and strengthens hydration, forming continuous water-rich pathways for transport (Figure 1B) [13,14,15]. These conformational transitions, which are contingent upon salt concentration, are intrinsically linked to the processes of ion pairing and dissociation, whereby the presence of solvated counterions, the local dielectric environment, and specific ion effects collaboratively influence both conductivity and segmental dynamics [16,17,18]. Tuning charge ratio and spatial distribution, side-chain chemistry, and crosslink density jointly control ion pairing versus external binding, local polarity and cation coordination, and the trade-off between mechanical stability and chain mobility for sustained ion transport (Figure 1C) [16]. In practical applications, the process of film formation may be impeded by pronounced internal ion pairing, which results in brittle, salt-like microstructures, thereby necessitating the development of composite matrices, dynamic or ionic crosslinks, and plasticized formulations that maintain percolated transport pathways while simultaneously enhancing material toughness [19].

Polyampholytes appear across electrochemical technologies as liquid and gel electrolytes, ionogels and eutectogels, binders, separators, and interfacial layers in batteries, supercapacitors, fuel cells, and solar cells (Figure 1D) [20,21,22]. Their performance is evaluated by ionic conductivity, cation transference number, electrochemical stability window, interfacial impedance, Coulombic efficiency, mechanical modulus and toughness, and the operating temperature range (Figure 1E) [23,24]. Dual charged networks are attractive because internal charge compensation reduces concentration polarization, promotes uniform ion flux, and enables safe and flexible operation over wide temperatures with good interfacial wetting. At the same time, device classes impose distinct constraints. Metal batteries face dendrite formation and unstable interphases, Li–S systems suffer shuttle, fuel cells require selective ion transport with minimal crossover, and aqueous devices must manage water activity and freezing. Some zwitterionic films are brittle, which complicates processing. Molecular and architectural remedies include hydrated zwitterionic channels, balanced charge architectures, dynamic or ionic crosslinks, plasticizers and ionic liquids, and nanofiber or inorganic reinforcement (Figure 1F) [25,26,27]. These strategies link chemistry and topology to transport, mechanics, and durability, setting up the structure–property–performance relations.

Recent reviews have significantly advanced the understanding of polymeric materials in electrochemical energy systems and provide the conceptual foundation on which the present work is built. Taghavi-Kahagh et al. [28] highlighted the broad potential of polyzwitterions in batteries, supercapacitors, solar cells, and fuel cells, clarifying how their coordination chemistry and mechanical response benefit energy storage. Safavi-Mirmahalleh et al. [29] elucidated how conductive hydrogels enable self-powered sensing and bioelectronic interfaces, thereby illustrating the importance of mechanical compliance and multifunctionality in soft ion-conducting networks. Li et al. [30] and Alam and Kumar [31] provided comprehensive overviews of polymers in flexible energy conversion and storage, mapping the diverse roles of polymers as electrodes, electrolytes, separators, and packaging layers. Complementarily, the review by Wang and Zhong [32] provided a broad overview of liquid, solid, and gel electrolytes for lithium-ion batteries, emphasizing safety, ionic conductivity, and electrolyte–electrode interfacial properties as key design parameters for next-generation energy-storage devices. Building on these contributions, the present review gathers and extends these ideas into a unified, mechanism-based framework focused specifically on polyampholytes, where charge ratio and spatial distribution, ion-pairing equilibria, anti-polyelectrolyte behavior, and salt-dependent conformational transitions are explicitly linked to macroscopic ion transport and interfacial performance across batteries, supercapacitors, fuel cells, and solar technologies.

Building on the conceptual foundations established by these prior studies, the present review consolidates dispersed insights into a coherent, chemistry-driven framework specifically tailored to polyampholytes. By bringing together principles from charge topology, hydration dynamics, ion-pairing equilibria, and polymer architecture, we outline how molecular-level design features translate into transport behavior, interfacial stability, and mechanical resilience across multiple electrochemical technologies. In contrast to broader discussions of polymer electrolytes, our analysis formalizes polyampholyte classification through parameters such as charge ratio, spatial distribution, monomer polarity, side-chain configuration, and crosslink density, enabling systematic structure–property–performance relationships to be identified across liquid, gel, ionogel, and eutectogel implementations. Integrating these molecular considerations with device-level requirements, including dendrite regulation, selective cation transport, antifreeze behavior, adhesion, and durability under strain, allows us to articulate design principles that can guide materials selection and engineering. In this way, the present review extends existing literature by unifying chemical, physical, and application-oriented perspectives and by outlining opportunities for next-generation polyampholyte architectures and scalable manufacturing pathways.

## 2. Chemistry, Classification and Design Strategies of Polyampholytes

Polyampholytes constitute a distinctive category of macromolecules characterized by the presence of both cationic and anionic functional groups integrated within the polymer backbone or its side chains, thereby engendering a multifaceted interplay of intramolecular and intermolecular electrostatic interactions that fundamentally dictate their physicochemical and electrochemical properties. The molecular architecture of these compounds, particularly the spatial configuration and proportionality of the oppositely charged moieties, is instrumental in influencing solvation dynamics, chain conformational behavior, ionic transport capabilities, and interfacial interactions within electrolyte systems utilized in advanced energy storage technologies, including batteries, supercapacitors, and solid-state devices [33,34,35]. In contrast to traditional polyelectrolytes which manifest predictable charge repulsion, polyampholytes demonstrate complex conformational transitions and anti-polyelectrolyte phenomena in aqueous and mixed solvent systems, thereby providing them with exceptional tunability regarding ionic conductivity, water retention, and mechanical robustness [36]. Recent investigations underscore that the deliberate molecular design of polyampholytes, accomplished through meticulous modulation of charge density, strategic selection of zwitterionic components, suitable polymer topology, and optimized crosslinking methodologies, facilitates precise regulation of ion coordination and mobility [37,38]. Consequently, a comprehensive understanding of the chemistry and design paradigms of polyampholytes is essential for leveraging their multifunctional capabilities as electrolytes, ion-conductive matrices, binders, and interfacial modifiers in electrochemical energy storage endeavors. This section delineates a framework for the classification and molecular engineering approaches pertinent to polyampholytes, while concurrently establishing the structure–property correlations that are pivotal to their emergent role as next-generation energy materials (Table 1).

Polyampholytes are typically categorized into three distinct classifications: annealed, quenched, and betainic (zwitterionic) systems, with this classification reflecting essential differences in the origin, distribution, and reactivity of ionic moieties along the polymer chain, which ultimately govern their physicochemical properties in solution and at interfaces [33,34,39,40]. Annealed polyampholytes are composed of weakly acidic and basic components, the degree of ionization of which is modulated by the surrounding environment; thus, variations in pH and ionic strength significantly affect protonation and deprotonation equilibria, resulting in adaptive charge densities, transitions from coil to globule conformations, and reversible electrostatic complexation with oppositely charged entities; this inherent responsiveness facilitates tunable ion transport while potentially undermining electrochemical stability under variable operating conditions [39,40].

**Figure 2:**
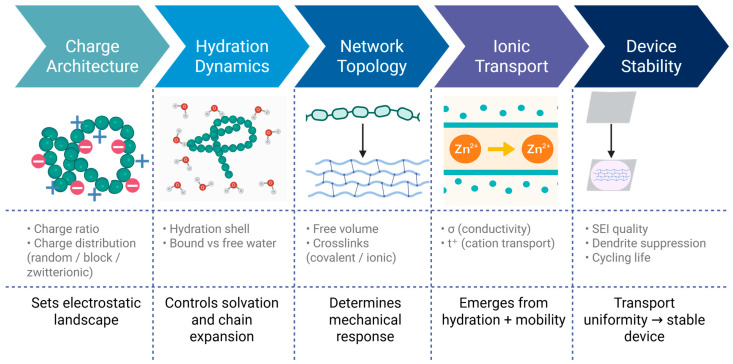
Mechanistic map of polyampholyte electrolyte design. Schematic overview illustrating how charge architecture, hydration dynamics, network topology, and ionic transport collectively determine device stability in polyampholyte-based electrolytes. Colored panels represent sequential design domains, where teal and blue tones indicate polymeric and aqueous environments, and orange symbols denote mobile charge carriers (e.g., Zn^2+^ ions). Green and teal bead-like structures represent polyampholyte chains containing oppositely charged or zwitterionic segments, while surrounding light-blue regions depict hydration shells and bound water. Solid arrows indicate causal or directional relationships, showing how molecular design parameters propagate from charge architecture through hydration and network topology to ionic transport and device-level performance. Horizontal arrows between panels denote mechanistic progression, whereas vertical arrows illustrate transport across electrolyte interfaces. Dotted grid lines separate conceptual layers and emphasize the multiscale linkage between molecular structure, ion conduction (σ and t^+^), and practical outcomes such as SEI quality, dendrite suppression, and cycling stability.

In contrast, quenched polyampholytes incorporate robust ionic groups into the polymer backbone or as pendant chains through covalent attachment that fixes their charge state, rendering their net charge independent of pH within the operational range. This covalent immobilization of ionic groups leads to non-responsive, strongly charged repeat units, electrostatic rigidity, enhanced structural integrity, and predictable ion-coordination behaviors that are primarily dictated by monomer sequence and macromolecular architecture rather than acid–base equilibria [33,40]. Betainic, or zwitterionic, polyampholytes are characterized by covalently bonded positive and negative charges within a single monomer unit. This molecular configuration yields an overall neutral charge and promotes extensive hydration through ionic solvation shells. A distinctive feature of these polymers is the anti-polyelectrolyte phenomenon, in which increasing salt concentration leads to chain expansion rather than contraction. This hydration-driven ion transport, together with suppressed nonspecific interactions, enhances their ion conduction efficiency. As a result, zwitterionic polyampholytes are increasingly employed as ion-conductive matrices, interfacial stabilizers and antifouling materials in electrochemical energy systems [41,42,43]. In this manner, annealed polyampholytes offer chemical flexibility, quenched polyampholytes deliver structural toughness, and zwitterionic polyampholytes encourage selective ion flow and interface reliability in both liquid and solid environments, thereby making the choice among these classes a critical design factor guided by the operational demands and performance standards of modern energy storage applications (Figure 2).

**Table 1:** Polyampholytes for energy storage.

Class	pH Responsiveness	Charge Mobility	Representative Polyampholytes	Key Energy-Relevant Features	Applications
Annealed	High	High (acid–base equilibria)	PAA–PAH ionic-bonded hydrogel (annealed weak acid/base) [42]	Regenerable, stretchable gel; pH-gated transport; robust flexibility	Flexible gel electrolytes; Zn-ion and Li-ion soft cells
			PVA–PAA hybrid GPE hierarchical porous network [44]	Hierarchical porosity; improved ion diffusion; mechanical integrity	Quasi-solid Li-ion cells; flexible devices
			PANa hydrogel (sodium polyacrylate) in alkaline Zn/air systems [45]	High stability in alkaline media; stable plating/stripping	Zn//NiCo, Zn-air; solid-state configurations
			PAA additive in aqueous Zn gel electrolyte [46]	Faster Zn^2+^ diffusion; uniform Zn deposition; lower polarization	Aqueous Zn-ion batteries
			P(AA-co-DMAEMA) and P4VP-co-MAA families (annealed PA archetypes) [47]	Coil–globule control; tunable complexation with cations	pH-responsive binders/separators; ion-gated gels
			PEI/PAA and PAH/PAA multilayer gels (annealed PA networks) [47]	Adjustable charge ratio; responsive swelling/porosity	Adaptive separators; gel binders
			Lean-water hydrogel concept applied in annealed matrices [48]	Wider ESW; fast transport with reduced water; safer operation	High-voltage Li-ion hydrogel cells
			Review evidence for hydrogel-based flexible supercapacitors [49]	Design rules for conductivity/elasticity trade-offs	Aqueous supercapacitors; all-in-one cells
Quenched	Low/none	Low (fixed ions)	APTAC–AMPS quenched PA hydrogels (strong cation + strong anion) [50]	pH-insensitive charges; stable mechanics; salt-tolerant swelling	Solid-like separators; mechanically stable gel electrolytes
			Equimolar PA AMPS–APTAC–DMAA networks (MDPI Polymers) [51]	Sequence/ratio control; tunable modulus; stable electrostatics	Separators, binders in aqueous cells
			Triple-network hydrogels with APTAC/AMPS (RSC JMC B) [52]	Architecture-driven toughness; controlled interfacial adhesion	Stretchable electrolytes; soft devices
			Quenched polyelectrolytes SANS/SAXS structure insights [53]	Hydrophobicity vs. charge fraction; pearl-necklace morphologies	Morphology control for ion pathways
			XLG/AMPS reinforced PA hydrogels [54]	Nanofiller reinforcement; rate-sensitive mechanics	Durable gel electrolytes under strain
			All-in-one flexible supercapacitor, solid-like hydrogel (design rules) [49]	Solid-like response; integrated electrode/electrolyte	Compressible supercapacitors
			Salt-tolerant amphoteric terpolymers AMPS–APTAC–AAm [55]	Stable swelling kinetics; tunable crosslink density	Separator matrices in saline/alkaline media
Zwitterionic	Minimal	Moderate (segmental motion; anti-PE)	Self-adhesive sulfobetaine/acrylamide hydrogel (SBMA/AAm) for ZIBs [56]	Lower interfacial resistance; long symmetric Zn cycling; adhesion	Flexible Zn-ion batteries; quasi-solid cells
			P(SBMA-co-BA):LiTFSI self-healing SPE for anode-free Li metal [57]	Self-healing via internal ion pairs; stable Li deposition	AFLMBs; solid-state Li cells
			Zwitterionic bottlebrush ionogels for Li^+^ transport [58]	Fast Li^+^ conduction; multifunctional damping; wide ESW	Solid polymer/ionogel electrolytes
			Anti-polyelectrolyte expansion in polyzwitterions (PNAS Nexus) [59]	Salt-induced chain expansion; enhanced hydration and mobility	Design lever for ionic conductivity in gels
			Zwitterionic brushes with anti-PE property [37]	Hydration shells; fouling resistance; ion selectivity	SEI-friendly interfaces; separators
			Conformal zwitterionic nanofilms stabilize Li metal [60]	Controlled SEI transport; dendrite suppression	High-energy Li metal anodes
			Zwitterionic PIL hydrogels with high transference for ZIBs [37]	Dendrite mitigation; stable Zn plating/stripping	Flexible ZIBs

GPE (gel polymer electrolyte); PANa (sodium polyacrylate); ESW (electrochemical stability window); ZIB (zinc ion battery); AFLMB (anode free lithium metal battery); SPE (solid polymer electrolyte); PA (polyampholyte); PAA (polyacrylic acid); PAH (polyallylamine hydrochloride); PEI (polyethylenimine); PVA (polyvinyl alcohol); P4VP (poly 4-vinylpyridine); DMAEMA (dimethylaminoethyl methacrylate); MAA (methacrylic acid); AMPS (2-acrylamido-2-methylpropane sulfonic acid); APTAC (3-acrylamidopropyltrimethylammonium chloride); DMAA (N,N-dimethylacrylamide); AAm (acrylamide); XLG (xanthan-like gum); PIL (polyionic liquid); SEI (solid electrolyte interphase); anti-PE (anti-polyelectrolyte effect); SBMA (sulfobetaine methacrylate); BA (butyl acrylate); LiTFSI (lithium bis(trifluoromethanesulfonyl)imide).

The rational molecular design of polyampholytes is fundamentally dependent on the synchronized regulation of charge equilibrium, hydration dynamics, monomer selection, side-chain chemical properties, and crosslinking methodologies, as these parameters collectively determine ion coordination, chain mobility, and interfacial compatibility within electrochemical systems. Primarily, the optimization of the density and arrangement of oppositely charged entities controls the balance between intra- and interchain electrostatic interactions, which subsequently governs hydration shell formation and the anti-polyelectrolyte phenomena that enhance ionic conduction in both aqueous and gel-based electrolytes [24,33,61,62]. Secondly, the chemical composition of monomers and the configuration of side chains define local polarity and hydrogen-bonding capacity, which in turn determine the cation-solvation structure and the extent of segmental motion essential for ion transport. Zwitterionic structures such as sulfobetaines and carboxybetaines generate well-defined hydration layers with low nonspecific adsorption [24,62]. Thirdly, the density and nature of crosslinking exert quantifiable control over the conductivity–mechanics trade-off: covalent networks increase modulus at the expense of segmental mobility, whereas ionic or dynamic crosslinks preserve elasticity and enable self-healing while maintaining continuous aqueous or plasticizer pathways for ion transport [24,33,61]. Ultimately, the strategic architectural decisions interconnect these variables at macroscopic scales, as linear (including block-sequenced variants), comb, and network configurations impose specific constraints on percolation and interfacial wetting phenomena. In practical applications, efficient designs for energy storage harmonize charge equilibrium with stable yet mobile hydration, select monomers and side chains that promote defined ion-solvation environments, and implement crosslinking strategies that preserve structural integrity without compromising dynamic behavior. This holistic approach to molecular engineering is foundational to the recent progress observed in polyampholyte electrolytes, binders, and separators, which successfully amalgamate high conductivity with interfacial stability in metal anodes and aqueous environments [24,33,44,61,62].

The charge ratio and spatial distribution significantly influence the structural characteristics, aggregation phenomena, and energetic profiles in polyampholytes. As the stoichiometric balance between cationic and anionic groups approaches equilibrium, the competition between intrachain pairing and ion pairing with salt leads to compact conformations under low ionic strength conditions, while higher salinity induces expansion, thereby illustrating the anti-polyelectrolyte effect prevalent in zwitterionic systems [33,61]. In contrast, intentional deviations from charge neutrality increase the net charge density, promoting extended conformations and enhancing long-range repulsive interactions, which facilitate ion accessibility but also increase osmotic swelling. Beyond net stoichiometry, the sequence of charge distribution is a major determinant of structure and dynamics because random arrangements of charged moieties generate heterogeneous local electric fields and broad distributions of loop and contact lengths, whereas block or gradient sequences create mesoscale domains that directly modify complexation dynamics and phase behavior [44]. This sequencing profoundly affects aggregation through selective screening mechanisms and can be strategically utilized to facilitate ion-rich pathways or to mitigate kinetically trapped aggregates that obstruct transport. Furthermore, the placement of charged groups alongside chains versus backbones modulates intercharge spacing and rotational freedom, thereby altering activation barriers for segmental motion and ion hopping. For energy-storage applications, modulation of charge ratio and distribution provides a mechanistically grounded strategy to reconcile conductivity with dimensional stability: nearly balanced, well-hydrated zwitterionic matrices minimize interfacial resistance, whereas slight charge imbalance or block-sequence structures can create continuous pathways for Li^+^ or Zn^2+^ without excessive swelling. Consequently, optimization of charge architecture should be performed together with salt concentration and solvent activity to position the system within well-defined regions of the transport–mechanics landscape [24,33,44,61,62,63].

Polymer architecture provides a direct way to couple local chemistry with mesoscale transport phenomena, and linear polyampholytes offer the simplest platform for examining how charge spacing and hydration influence coil dimensions and ion mobility. Within this linear category, block copolymers constitute a sequence-defined subclass in which chemically distinct segments are arranged in ordered blocks rather than statistical distributions. This distinction reflects monomer sequence rather than a separate polymer topology. Block-copolymer architectures create microphase-separated domains that place ionic groups into continuous transport regions and position mechanically robust segments elsewhere, which supports simultaneous stiffness and ion mobility in solid or gel states [44,64]. Comb and brush architectures further enhance this decoupling because ion-solvating side chains are anchored to an immobile backbone, and the resulting increase in free volume and reduction in entanglement promote higher segmental dynamics at a constant modulus, consistent with reports on zwitterionic brushes that show salt-responsive swelling and low interfacial resistance [24,62]. Network and interpenetrating-gel architectures translate architectural control into improved device resilience, since crosslink topology determines crack resistance, self-healing behavior, and electrolyte retention, and dynamic or ionic crosslinks help maintain conductivity under strain while preserving structural integrity. Hyperbranched and bottlebrush-like topologies suppress crystallinity and entanglement and generate percolated nanochannels that enhance ion transport, while dense side-chain coronas stabilize hydration and limit parasitic adsorption at interfaces [24,44,62,63]. Consequently, polymer architecture functions as a critical design variable that governs the formation of ion-rich pathways, the dissipation of mechanical stress during cycling, and the long-term stability of electrode–electrolyte interfaces, and optimal performance emerges when architectural choices are aligned with the target ion, solvent activity, and mechanical demands of the device [24,33,44,61,62,63].

## 3. Functional Properties Relevant to Energy Storage

### 3.1. Physicochemical, Mechanical, and Self-Healing Attributes Governing Ion Transport

Anionic and cationic groups whose intra and intermolecular electrostatic interactions govern solubility, chain conformation, and coil to globule transitions, thereby shaping ion transport in devices [6]. In dilute salt free media, like charges expand conventional polyelectrolyte coils, while added electrolyte screens repulsions and lowers viscosity, the classic polyelectrolyte effect [52]. By contrast, many polyzwitterions display salt-induced chain expansion, the anti-polyelectrolyte effect, which depends on salt identity and concentration and promotes hydration and continuous water rich domains that facilitate conduction [53,54]. Because internal charge compensation suppresses concentration polarization at the electrode–electrolyte interface, polyampholytes help equalize ion distribution, minimize local voltage drops, and improve reversibility during fast cycling [37,59]. Although some zwitterionic matrices are brittle, hydrophilic reinforcements such as cellulose nanofibers increase water retention and flexibility, creating interpenetrating microporous networks that stabilize transport pathways without sacrificing mechanical integrity [55,56]. Consistently, grafting zwitterionic groups onto CNF modified polyurethane yields hydrogels with high toughness, rapid self-healing, and substantial conductivity even at subzero temperature, attributed to non-freezable water formed by electrostatic interactions and dynamic Zn^2+^ coordination [57]. Mechanically, reversible ionic pairing between opposite charges acts as physical crosslinks that dissipate stress and enable autonomous recovery, with performance emerging from a balance of charge ratio, chain mobility, swelling, and crosslink density [24,60,61]. Dual network and gradient architectures further raise resistance to deformation while preserving segmental dynamics, and cyclic loading often reveals nonlinear hardening and hysteresis consistent with repeated opening and closing of ionic bonds [33,62]. Water and environmental stimuli modulate these processes, since moisture plasticizes chains and pH or salt adjust ionization and pairing, which together tune healing kinetics and steady ion flux [44,58].

### 3.2. Interfacial Compatibility and Solid Electrolyte Interphase Regulation

Polyampholyte ionic groups govern not only bulk transport but also electrode–electrolyte compatibility and interphase chemistry, which together control reversibility, rate capability, and safety [38]. Zwitterionic solid and gel electrolytes form transient, field-aligned migration channels while maintaining charge neutrality; their labile ion–ion associations support both vehicular motion and structural hopping, thereby sustaining high conductivity without sacrificing interfacial order [63,64]. In aqueous Zn systems, sulfobetaine–cellulose hydrogels guide Zn^2+^ to deposit uniformly by combining hydrated, water-rich domains with electrostatic adhesion at the metal surface, which lowers interfacial resistance, suppresses concentration polarization, and mitigates dendrite nucleation [14,38,65,66,67,68]. Consistently, these matrices promote a homogeneous quasi-SEI that remains ionically conductive; spectroscopy and impedance data corroborate a stable, mainly organic interphase that supports smooth plating and stripping over extended cycling [38,68,69]. Beyond hydrogels, zwitterionic salts and ionic-liquid hybrids widen transport corridors while preserving electroneutrality, improving ion dissociation, and evening the electric-field distribution at the interface, which together reduce overpotentials and enhance rate performance [65,68,69]. Under an applied field, ordered zwitterionic side groups create preferential cation and anion pathways that lessen ion pairing, maintain steady-state flux, and limit gradient buildup near the interface, benefits that are especially evident in high-rate supercapacitors and Zn batteries [70,71]. Finally, by fostering controlled SEI or quasi-SEI formation and robust wetting across temperatures, polyampholytes improve cyclability and reduce safety risks associated with interfacial instability in both aqueous and non-aqueous cells [72,73].

A comparative assessment of representative polyampholyte electrolytes highlights how their interfacial behavior correlates with measurable transport and mechanical parameters (Table 2). Systems that combine strong hydration shells with mobile ionic crosslinks tend to exhibit higher ionic conductivity and improved cation transference, aligning with their capacity to maintain uniform ion fluxes at reactive metal surfaces. Electrolytes with balanced charge distributions and zwitterionic architectures typically support broader electrochemical stability windows and generate more homogeneous SEI or quasi-SEI layers, consistent with reduced overpotentials and smoother plating–stripping profiles [38,74,75,76,77,78,79,80,81]. Mechanical characteristics such as toughness and elasticity also align closely with interfacial performance, as tougher networks better resist morphological disruption during cycling, while flexible matrices accommodate volume changes without compromising interphase integrity. Collectively, these trends underscore that interfacial compatibility in polyampholyte systems emerges from the coordinated interplay between molecular mobility, hydration structure, charge topology, and mechanical resilience.

## 4. Applications in Electrochemical Devices

Polyampholytes address key bottlenecks in electrochemical systems by coupling high ionic mobility with interfacial stabilization, mechanical compliance, and safety. Their dual charge enables selective ion coordination, hydration regulation, and internal charge compensation, which together enhance conductivity, suppress parasitic reactions, and improve cycling stability across batteries and supercapacitors [19,35,45,72,73,74,75,76].

### 4.1. Polyampholytes in Batteries

Zwitterion-based gels and solid polymer electrolytes regulate Li^+^ solvation and raise transference while preserving robust interfaces (Table 3) [74,75,76,77,78,79,80,81,82,83,84]. A polyampholyte-derived artificial interphase reached t^+^(Li^+^) = 0.81 with σ = 7.5 × 10^−5^ S cm^−1^, enabling stable Li plating/stripping for ~1400 h at 1 mA cm^−2^ [81]. Incorporating a flexible segment (G4) with carboxybetaine methacrylate balanced Li^+^/anion distributions, enabling symmetric Li‖Li cycling for more than 5500 h at 30 °C and delivering 99.9% capacity retention over 320 cycles in Li‖LFP cells and 62.5% retention after 54 cycles in Li‖NCM811 cells [82]. Dual-network electrolytes that polymerize zwitterions concurrently with a non-hydrolytic sol–gel silica phase delivered σ = 0.44 mS cm^−1^ at 30 °C, an electrochemical window > 5 V, tensile strength 0.75 MPa, and 560% elongation [83]. Zwitterion-cross-linked ionogels disrupted Li^+^–TFSI^−^ clustering, increasing σ(Li^+^) from 0.23 to 0.44 mS cm^−1^ and t^+^ from 0.23 to 0.37 at room temperature, with good low-temperature capability [38]. For Li–S, an imidazolium-type polyzwitterion grafted on lithiated Nafion (ZIGLN) provided σ = 1.35 × 10^−3^ S cm^−1^, t^+^ = 0.75, long-term low-ΔV symmetric stability, and 460 mAh g^−1^ at 1 C with shuttle suppression [84].

Polyzwitterionic hydrogels furnish high hydration, strong interfacial adhesion, and uniform Zn^2+^ flux while reducing hydrogen evolution and dendritic growth. PSBMA hydrogels showed σ = 32 mS cm^−1^ (25 °C) and 83.5 mS cm^−1^ (95 °C) with ~2000 h Zn|Zn stability and CE ≈ 99% [74]. A METAC–NaSS copolymer hydrogel achieved σ = 27.3 mS cm^−1^, 600 cycles at 5 A g^−1^, and self-healing when reinforced, indicating robust ion pathways and adhesion [75]. Zwitterionic hydrogel networks improved conduction and toughness with 600 cycles at 5 A g^−1^ [79]. A quasi-solid PSBMA-based gel in Zn(ClO_4_)_2_ formed a gradient interphase, giving σ = 6.48 mS cm^−1^, 240 mAh g^−1^, and CE = 99.18%, with 1000 cycles in Zn|Cu cells [77]. Antifreeze PSBMA hydrogels retained 1.6 mS cm^−1^ at −21 °C and 34.8 mS cm^−1^ at 80 °C, delivering 154.1 mAh g^−1^ over 500 cycles [78]. As an electrolyte environment, PSBMA equalized fields and suppressed dendrites, enabling ~3500 cycles and 363.1 mAh g^−1^ [79]. A phosphate-bearing polyampholyte (PMEAP) supported 270 mAh g^−1^, CE ≈ 99%, and 10,000 cell cycles [80]. To limit water-driven side reactions while preserving transport, a zwitterion-based DES eutectogel delivered σ = 7.1 mS cm^−1^ (20 °C), a wide electrochemical window, >2200 h Zn|Zn, and 73.9% capacity retention over 500 V_2_O_5_ cycles, with thermal mass retention at 60 °C [81]. Protective anodes are also effective in stabilizing zinc deposition. A water-blocking P(SBMA-co-BuA) coating maintained Zn|Zn cycling for 2500 h at 1 mA cm^−2^ and supported 3500 full-cell cycles at 5 A g^−1^ without short circuiting [79]. A phosphorylcholine-based interphase (PZIL) further improved anode stability by leveraging Zn^2+^ chelation and Hofmeister effects, enabling more than 1000 h of symmetric cycling at current densities up to 40 mA cm^−2^ while maintaining high Coulombic efficiency and stable polarization [80].

Zwitterion-rich hydrogels facilitate the equalization of interfacial fields, maintain elevated water activity without the occurrence of uncontrolled side reactions, and prolong the lifetimes of symmetric cells from hundreds to thousands of hours, despite variations in electrolyte chemistries and testing temperatures [38,74,75,76,77,78,79,80,81]. PSBMA matrices exhibit room-temperature conductivities ranging from 1.6 to 32 mS cm^−1^ and preserve functionality at both subzero and elevated temperatures, thereby elucidating their exceptional performance as quasi-solid electrolytes and binders [74,78]. Eutectogels formulated from deep eutectic solvents (DESs) compromise some room-temperature conductivity in exchange for broader electrochemical windows and minimized parasitic reactions, which empower extended symmetric cycling and robust full-cell performance [81]. Coating methodologies extend this design rationale to the interface: zwitterionic layers that either block water or chelate ions effectively modulate Zn^2+^ flux, mitigate hydrogen evolution, and enable stable operation at elevated current densities [79,80]. The underlying mechanistic principle is unequivocal: the balance of charge-neutral hydration and robust ion–dipole interactions engenders percolated, water-rich pathways that standardize deposition while preserving dimensional integrity. The associated limitations are equally evident. Numerous studies on zinc report cell longevity in symmetric configurations but offer minimal data on transference numbers, electrochemical stability windows (ESW), or tests concerning high areal capacity, which complicates cross-study benchmarking and the translation of findings to practical pouch designs [38,74,75,76,77,78,79,80,81]. The standardization of electrode areal loading, electrolyte thickness, and comprehensive reporting of t^+^ and interfacial resistance would enhance the mapping of structure–performance relationships.

In lithium systems, three complementary pathways are identifiable. Initially, polyampholyte-derived artificial interphases enhance Li^+^ transference to approximately 0.8 at moderate conductivity levels, thereby stabilizing plating during prolonged dwell times, which is advantageous in rate-limited or low-electrolyte regimes [81]. Subsequently, zwitterion-rich bulk electrolytes achieve extended lifetimes in symmetric cells and robust full-cell cycling when segmental mobility is introduced through flexible spacers or dual networks, with the inorganic sol–gel phase concurrently enhancing anodic stability and mechanical reinforcement [82,83]. Lastly, ionogels wherein zwitterions disrupt Li^+^–TFSI^−^ clustering increase Li^+^ conductivity and transference numbers at room temperature, suggesting a broadly applicable strategy for the de-pairing of ions and selective cation transport [38]. In lithium–sulfur systems, zwitterionic separator coatings facilitate the capture of polysulfides while guiding lithium nucleation, thus improving both shuttle suppression and dendrite control within a unified architecture [84]. The potential lies in the intentional co-optimization of ion dissociation, chain mobility, and interfacial chemistry within a single compositional platform, rather than addressing these factors in isolation. Remaining challenges encompass a lack of operando evidence correlating local solvation to macroscopic transference numbers, limited cycling performance at high cathode areal loadings, and inadequate reporting on thickness-normalized impedance and low-temperature kinetics [38,81,82,83,84]. Addressing these issues through harmonized protocols and in situ measurements would transform current proof-of-concept findings into transferable design principles for scalable energy storage systems.

### 4.2. Polyampholytes in Supercapacitors

Supercapacitors benefit from polyampholyte electrolytes that couple high ionic conductivity with elasticity and subzero operability (Table 4). Proline-based zwitterionic hydrogels sustain 4.2 mS cm^−1^ at −40 °C while delivering 145.8 mF cm^−2^, indicating sustained ion transport at subzero temperature [85,86]. Zwitterion-rich PSBMA networks that promote LiCl dissociation reach 12.6 mS cm^−1^ at −40 °C and maintain capacitance after prolonged low-temperature exposure, functioning simultaneously as strain sensors due to intrinsic adhesion [87]. Random copolyampholytes achieve 13.2 mS cm^−1^ with low charge-transfer resistance and preserve 87.1% capacitance after 10,000 cycles, highlighting the role of charge balance and network dissipation in durability [88]. Acid-doped, BAGU-cross-linked polyampholytes retain 6.88–8.23 mS cm^−1^ from −30 to 20 °C and withstand freeze–thaw cycling, demonstrating that hydrogen-bond immobilization of free water is an effective anti-freezing design strategy [89]. Adhesive quasi-solid gels that combine anionic and cationic functionalities deliver strong binder-like contact, 15 mS cm^−1^ at −20 °C, 241 F g^−1^, and 92% retention after 10,000 cycles, underscoring interfacial mechanics as a co-determinant of electrochemical stability [90].

Performance can be further increased by molecular and formulation strategies. Re-dox-assisted zwitterionic gels increase specific capacitance from the 100–200 F g^−1^ range to 677 F g^−1^ and support multi-volt operation with energy and power densities up to 542 Wh kg^−1^ and 2.88 kW kg^−1^, reflecting added faradaic contributions without sacrificing gel integrity [86]. Synergistic pairing of polyzwitterions with ionic liquids enhances ionic mobility and capacitance retention, as shown by VIPS–IL systems that deliver 108.8 F g^−1^ with 99% retention after 1000 cycles [91]. Double-network polyzwitterionic electrolytes loaded with EMIM-BF_4_ widen the temperature window to −60 to 50 °C, raise tensile strength to 1.9 MPa, and maintain 7.24 mS cm^−1^, illustrating that high mechanical strength and fast ion transport can be co-optimized [92]. All-hydrogel integrated devices that use the same polyampholyte as both electrode matrix and electrolyte achieve compressible, self-adhesive architectures with stable areal capacitance suitable for wearables [93]. For context, benchmark IL electrolytes establish wide-voltage-window baselines [94], while IL composites and IL–solvent hybrids improve wetting and achievable energy density [95,96,97,98,99]. Salt-in-IL hydrogels provide cold-resistant ion transport down to −50 °C with cycle retention typically above 90%, offering a complementary path to subzero performance [100,101].

Across Table 3, three pivotal design levers systematically elucidate performance outcomes: bound hydration, ionic-liquid (IL) synergy, and network architecture. Initially, antifreeze zwitterionic hydrogels leverage non-freezable water to maintain the activity of ion pathways: proline-based gels exhibit conductivity of 4.2 mS cm^−1^ at −40 °C, accompanied by functional areal capacitance, while PSBMA networks attain a conductivity of 12.6 mS cm^−1^ at −40 °C, sustaining approximately 95% capacitance over a duration of 30 days, thereby substantiating the concept of hydration-shell engineering as the preeminent strategy for sub-zero operational efficacy [85,87]. Secondly, redox-assisted electrolytes can increase apparent capacitance by introducing faradaic contributions. For example, the combination of PPDE, LiCl, and ethyl-viologen yields a capacitance of 677 F g^−1^ and provides energy and power densities of 542 Wh kg^−1^ and 2.88 kW kg^−1^, respectively. These values cannot be directly compared to double-layer systems because they represent hybrid behavior rather than true EDLC enhancement [86]. Thirdly, IL coupling expands both temperature and voltage operational windows while simultaneously maintaining material softness: zwitterion–IL systems exhibit ionic conductivities on the order of 10^0^–10^3^ mS cm^−1^ together with tensile strengths up to 1.9 MPa and stable capacitance retention across −60 to 50 °C (for instance, VIPS/SPMA and VBIPS/EMIM-BF_4_ demonstrate 7.24 mS cm^−1^ conductivity and 1.9 MPa mechanical strength, functioning across the temperature range of −60 to 50 °C), while benchmark PYR_14_TFSI provides a contextual reference for realistic EDLC performance [91,93,94]. Moreover, adhesive polyampholyte matrices further facilitate the development of all-hydrogel devices characterized by reliable interfacial contact and areal capacitances on the order of 100–250 mF cm^−2^ with ≥90% retention after long-term cycling, which are essential prerequisites for wearable applications [90,93].

Despite the notable achievements in point metrics, several limitations persist. The reported conductivities and capacitances encompass various forms of normalization (areal versus gravimetric), temperature ranges, and device architectures, thereby complicating direct benchmarking comparisons; future investigations ought to engage in pairing EDLC controls (for example, PYR_14_TFSI) with uniform electrodes and thicknesses to effectively isolate the effects of electrolytes [94]. From a mechanical perspective, enhanced double-networks and acid-doped gels demonstrate improved freeze–thaw resilience (for example, 6.88–8.23 mS cm^−1^ maintained from −30 to 20 °C with capacitance retention of 97.01% at 20 °C and 45.77% at −30 °C after 500 cycles), yet they may limit segmental mobility, thus rendering the optimization of crosslink density for simultaneous strength and ion transport a central concern [89]. Ionogels and IL/solvent hybrids elevate energy density (reaching approximately 50–60 Wh kg^−1^) while preserving flexibility; however, challenges related to wetting, long-term chemical stability, and leakage management continue to impede scalability [95,96,97,98]. Lastly, “salt-in-IL” hydrogels facilitate cold-resistant transport capabilities down to −50 °C with capacitance retention typically in the 92–100% range depending on the salt/solvent combination, but comprehensive assessments concerning broader abuse testing, humidity resilience, and durability under bending and rolling conditions remain insufficiently reported [99,100,101,102,103,104,105]. Collectively, the most salient avenues for advancement within the field are: standardization of performance metrics; decoupling mechanical strength from mobility through hierarchical or double-network architectures; utilizing zwitterion–IL synergy to extend operational windows; and innovatively designing intrinsically adhesive, non-freezable networks that maintain capacitance under realistic mechanical loading conditions [105,106,107,108,109].

## 5. Current Limitations and Engineering Approaches for Polyampholyte-Based Electrochemical Materials

Recent advances highlight the promise of polyampholytes in next-generation electrochemical systems, yet several intrinsic limitations continue to constrain their practical deployment. A primary challenge arises from the strong Coulombic interactions between oppositely charged groups, which promote the formation of ionically associated microdomains rather than fully dissociated polymer networks. These microdomains behave as physical crosslinks and often generate brittle hydrogel films under processing or environmental fluctuations [18,28,32,41,48]. Such brittleness reduces deformation tolerance, induces cracking, and compromises cycling reliability under repeated mechanical or thermal stress. Strong ion pairing and dense charge compensation can also suppress segmental mobility and reduce effective ion diffusivity, particularly in tightly crosslinked or salt-rich formulations [28,105]. In wearable and biomedical devices, further constraints appear, including the need for non-toxic components, self-healing behavior, and durable electrode–electrolyte adhesion under deformation [105,108,109]. These factors collectively define a multi-parameter design space in which charge topology, hydration structure, crosslink density, and polymer architecture must be balanced to avoid tradeoffs between conductivity, stability, and mechanical resilience.

**Figure 3:**
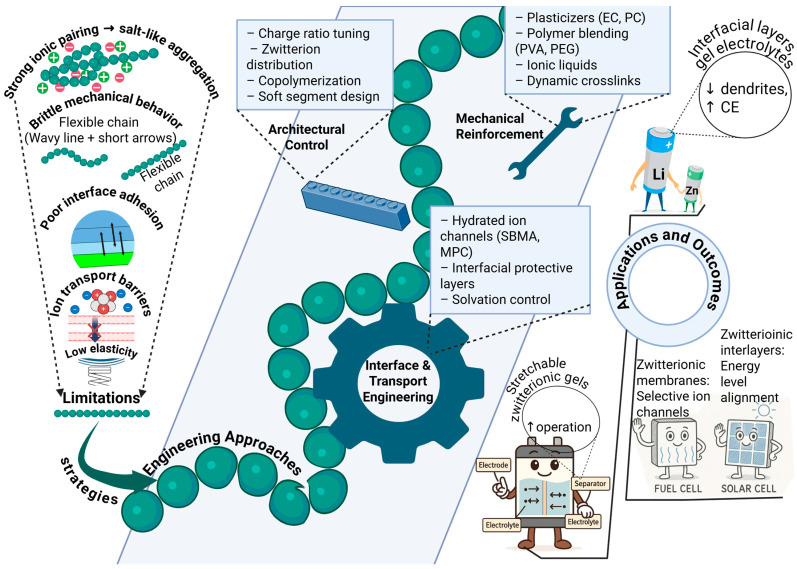
Engineering strategies to overcome limitations of polyampholytes in electrochemical energy systems. Schematic illustration of molecular, architectural, and interfacial engineering approaches used to simultaneously optimize ion transport, mechanical robustness, and interfacial stability. Green bead-like chains represent polyampholyte backbones containing oppositely charged and zwitterionic segments, while blue shaded regions indicate functional domains involved in mechanical reinforcement or transport control. White boxes summarize specific design strategies, including charge ratio tuning, zwitterion distribution, copolymerization, soft-segment design, plasticization, polymer blending, ionic liquids, and dynamic crosslinking. Solid arrows denote directed ion transport or structure–property optimization pathways, whereas dashed arrows indicate conceptual links between architectural control, interface engineering, and performance outcomes. Gear and wrench symbols represent interfacial/transport engineering and mechanical reinforcement, respectively. Application icons illustrate representative electrochemical systems (Li- and Zn-based batteries, fuel cells, and solar cells) enabled by these strategies.

To address these limitations, engineering strategies increasingly aim to decouple mechanical robustness from ionic transport. Incorporation of plasticizers such as propylene carbonate and ethylene carbonate reduces chain packing density and disrupts strong ionic associations, thereby improving flexibility without entirely compromising ion conduction (Figure 3). Polymer blending with flexible backbones such as PVA, PEGMA, and PEO introduces segmental mobility that dissipates mechanical stress while supporting ion movement. A notable example is the VBIPS–acrylamide copolymer crosslinked with PVA and EMIM-BF_4_ ionic liquid, which exhibits exceptional recovery after deformation and a mechanical resistance twenty times higher than the water-based analogue [96]. Complementary advances demonstrate that more sophisticated molecular design can convert strong ionic interactions from a drawback into a structural advantage. Imidazolium–sulfonate dimeric bonding has produced polyampholyte elastomers with tensile strength above 20 MPa, a modulus above 200 MPa, and lithium conductivities near 10^−3^ S cm^−1^ [102]. Likewise, hydrogels with structured hydration layers formed through self-assembly of balanced cationic and anionic monomers stabilize interfacial water, enhance energy dissipation, and provide both high elasticity and robust mechanical performance [106]. These findings collectively indicate that hierarchical ionic crosslinking and controlled hydration represent powerful routes for mitigating brittleness while maintaining electrochemical function.

Beyond mechanical stability, rational architectural design plays a central role in optimizing ion transport, interfacial chemistry, and electrochemical performance. In rechargeable batteries, carefully engineered distributions of anionic and cationic groups help maintain balanced internal electric fields and strong interfacial adhesion, both of which are critical for suppressing dendrite formation in lithium and zinc systems [15,65]. Introduction of zwitterionic units such as sulfobetaine or phosphorylcholine creates hydrated channels that regulate solvation structure and reduce parasitic side reactions at electrode interfaces [14,96]. In zinc-ion devices, eutectogel architectures composed of polyampholytes and ionic liquids provide enhanced compatibility with zinc anodes and long-term cycling efficiencies exceeding 2000 h [14,38]. In lithium systems, polyampholyte gel electrolytes and interfacial films modulate Li^+^ solvation environments, stabilize electrode interfaces, and increase Coulombic efficiencies to >98–99% [17,77]. At the level of redox-active polymers, covalent attachment of hydroquinone to polyallylamine suppresses quinhydrone formation through electrostatic repulsion, enabling reversible and thorough charge storage in aqueous batteries [107]. These examples highlight that local coordination chemistry, hydration structure, and polymer segment mobility are key levers for achieving high conductivity and stable electrode interfaces.

In supercapacitor systems, the dual-charged character of polyampholytes provides additional tunability of ion transport and electrostatic interactions at electrode interfaces. Rational choice of zwitterionic groups enables stable double-layer formation with minimal resistive loss and predictable ion selectivity [34,45]. Zwitterionic motifs such as sulfobetaine and phosphorylcholine provide exceptional antifreeze performance by suppressing ice crystallization, enabling sub-zero operation in hydrogel electrolytes [88,94,97,98]. Incorporation of ionic liquids or redox-active zwitterionic components allows hybrid storage mechanisms that combine double-layer and pseudocapacitive behavior. Recent demonstrations show ionic conductivities exceeding 10 mS·cm^−1^ and capacitance retention above 85–95% during extended cycling, even under repeated mechanical deformation [105]. Biocompatible polyampholyte hydrogels swollen with simulated body fluid also achieve high conductivities (~17 mS·cm^−1^ at 35 °C) with negligible electronic leakage and high areal capacitance in carbon-based supercapacitors, further showing compatibility with implantable devices [107]. These studies illustrate the broad chemical and architectural tunability available for enhancing supercapacitor performance.

In solar energy conversion and fuel cell technologies, zwitterionic interlayers play specialized roles in tuning interfacial energy levels, suppressing recombination, and improving ion selectivity. In organic and perovskite solar cells, oriented zwitterionic dipoles reduce electrode work functions and enhance charge extraction, while conjugated backbones help maintain electronic conductivity [25,38,84,96]. In fuel cells, zwitterionic polymers must simultaneously conduct protons or hydroxide ions and block fuel molecules. Incorporation of sulfobetaine groups into polymer or MOF-based membranes promotes selective hydrogen-bond-mediated transport while reducing methanol crossover [34]. Integration with porous hosts such as ZIF-8 further improves ion selectivity. Future work must enhance long-term operational stability and enable scalable membrane fabrication suitable for industrial deployment.

Finally, sustainability and application-specific constraints are emerging as important design considerations. Cellulose-derived hydrogels offer biodegradable, biocompatible alternatives to conventional superabsorbent polymers while achieving competitive swelling and retention properties [104]. When combined with zwitterionic or ampholytic motifs, such systems can support both environmental sustainability and electrochemical performance. Polyampholyte hydrogels designed for wearable and implantable devices already show high conductivity, self-healing, and low cytotoxicity under physiological conditions [105,106,109]. Beyond these advances, future work must also evaluate synthetic scalability. Although controlled RAFT methods enable precise tuning of charge ratio, sequence, and side-chain architecture, translation to industrial production will require comparison with scalable aqueous polymerization routes that use benign solvents, lower cost reagents, and simplified purification. RAFT-based tools should therefore complement, rather than replace, high-volume aqueous free-radical polymerizations, with each pathway addressing different performance–manufacturing tradeoffs [103].

Compatibility with next-generation battery architectures also represents an important future challenge. In particular, polyampholyte gel electrolytes and interfacial layers must maintain chemical and mechanical stability when paired with high-loading cathodes (≥5 mAh cm^−2^), where large ion fluxes and electrode expansion place additional demands on transport homogeneity and interface adhesion. Evaluating polyampholyte networks under these practically relevant conditions will be essential for translating laboratory performance into device-scale operation [17,77].

At lithium metal anodes, another critical direction involves clarifying the interplay between polymer segment mobility and solid–electrolyte interphase (SEI) crystallinity. Polyampholyte networks may either promote or hinder the formation of uniform, flexible SEI phases depending on their solvation behavior and local ionic microstructure. Understanding how polymer mobility, dipole strength, and hydration motifs influence SEI composition and crystallinity will guide the molecular design of safer Li-metal interfaces. These examples underscore the need for future engineering approaches that integrate molecular design, device architecture, and life-cycle considerations so that polyampholyte-based materials can meet the performance, safety, and regulatory requirements of real-world electrochemical technologies [22,37,81].

## 6. Conclusions

Polyzwitterions and related polyampholytes have emerged as versatile soft ionic conductors and interfacial modifiers that address several chronic bottlenecks in electrochemical energy devices. Across batteries, supercapacitors, solar cells, and fuel cells, their internal charge balance reduces concentration polarization, smooths ion flux, and enables formation of uniform passivating layers that resist dendrites and parasitic reactions. Hydration control, ion–dipole coordination, and dynamic ionic clustering collectively raise conductivity while preserving mechanical integrity when properly formulated. In aqueous and quasi solid environments, these polymers stabilize water rich domains that support low temperature conduction, whereas in organic and ionic liquid media they promote salt dissociation and selective cation transport. As a result, devices gain in rate capability, cycle stability, and safety, while also gaining new functions such as self-healing and strong adhesion to electrodes.

For batteries, the most consistent benefits arise at metal anodes and separator or electrolyte interfaces. In zinc systems, zwitterionic gels and eutectogels regulate Zn^2+^ solvation, homogenize the interfacial field, and markedly suppress dendrite nucleation, which extends symmetric cell lifetimes and stabilizes full cells under high-current operation. In lithium systems, polyampholyte-derived artificial interphases and zwitterion-rich gels increase Li^+^ transference numbers, reduce overpotential by quantifiable margins reported in the literature (typically 50–150 mV depending on current density), and protect highly reactive surfaces during plating and stripping. Dual-network polymer electrolytes and ionogels that integrate zwitterionic segments with inorganic or ionic-liquid scaffolds achieve a robust balance of strength and conductivity and open wider electrochemical windows. In lithium–sulfur cells, zwitterion functional layers on separators limit polysulfide migration while guiding Li^+^ nucleation, which improves Coulombic efficiency to values commonly exceeding 98–99% and extends cycling. Together, these results demonstrate that rational charge architecture and tailored solvation environments can convert fragile interfaces into durable, highly conductive ones.

In supercapacitors, polyampholyte hydrogels function as electrolyte, separator, and binder in compact formats that remain conductive over large temperature ranges and after repeated deformation. Antifreeze behavior is achieved by binding free water and by strengthening hydrogen bonding networks, which sustains ionic conductivity at subzero temperature and enables wearable devices. Redox additives and zwitterionic poly(ionic liquid) frameworks further boost energy density while preserving fast charge–discharge. In optoelectronic devices, zwitterionic interlayers lower electrode work functions through oriented interfacial dipoles, improve carrier extraction, and stabilize perovskite or organic films during operation. Early results in fuel cells show that zwitterion modified membranes and MOF-based channels can combine cation and anion conduction while limiting fuel crossover. These cross domain advances highlight the unifying role of zwitterionic chemistry in coupling ion transport with mechanical resilience and interfacial control across disparate device platforms.

Key challenges remain and create a clear agenda for materials design and translation. Many polyzwitterion films are brittle and require molecular softening through flexible co monomers, dynamic crosslinks, plasticizers such as ionic liquids or carbonates, or nanofiber reinforcement without sacrificing transport. Ion pairing and strong, unintended complexation with multivalent cations (for example, Zn^2+^, Mg^2+^) can throttle conductivity, which motivates side-chain engineering, counterion selection, and precise control of charge ratio and distribution. Future efforts should integrate operando characterization of solvation, interphase growth, and mechanical dissipation with multiscale simulation and data-driven discovery to map design rules that transfer across chemistries and devices. Attention to manufacturability, toxicity, recyclability, and cost will be essential for scaling. Overall, polyampholytes offer a coherent strategy to unite fast ion conduction with stable interfaces and soft mechanics.

## Figures and Tables

**Table 2:** Benchmark comparison of representative polyampholyte electrolytes.

System/Device	Polyampholyte Electrolyte	σ (S·cm^−1^)	t^+^	Electrochemical Stability Window (ESW, V)	Mechanical Toughness	Temperature Window (°C)	Ref.
Li metal cell	Polyampholyte-derived artificial interphase	7.5 × 10^−5^	0.81 (Li^+^)	n.r.	Stable Li plating/stripping for ~1400 h	~25	[38,74,75,76,77,78,79,80,81]
Li-ion solid electrolyte	Dual-network polyzwitterion + inorganic TEOS network	4.4 × 10^−4^	n.r.	>5	Tensile strength 0.75 MPa; elongation 560%	~25	[82,83]
Li-ion ionogel	Zwitterion-cross-linked ionogel (pCBMA-based)	(2.3–4.4) × 10^−4^	0.23–0.37	n.r.	Soft, flexible ionogel; crack-resistant	Room to low-T	[38]
Zn-ion battery	PSBMA hydrogel electrolyte	3.2 × 10^−2^	n.r.	Aqueous, n.r.	Tough hydrogel; stable Zn‖Zn cycling ~2000 h	25–95	[74]
Stretchable supercapacitor	VBIPS/EMIM-BF_4_ double-network ionogel	7.24 × 10^−3^	n.r.	Wide multi-volt range	Tensile strength 1.9 MPa; stretchable	−60 to 50	[84,85,86,87,88,89,90,91,92]
Biocompatible supercapacitor	Polyampholyte–simulated-body-fluid (SBF) hydrogel	1.76 × 10^−2^ (35 °C)	n.r.	Aqueous, n.r.	Flexible, self-healing hydrogel	~35	[93,94]

n.r. = not reported explicitly in the cited work.

**Table 3:** Polyampholyte-based components in rechargeable batteries.

Battery System	Polyampholyte (Abbrev.)	Role in Cell	Medium/Formulation Highlights	Key Electrochem. Metrics (σ, t^+^, Window)	Test Rate/Current Density	Cycle Life & Stability	Capacity/CE/Overpotential	Temp. Window	Headline Effects	Ref.
Zn-ion (aqueous)	PSBMA hydrogel	Gel electrolyte/binder	High hydration; dual-charge network stabilizes water-rich domains	σ: 32 mS cm^−1^ (25 °C); 83.5 mS cm^−1^ (95 °C)	1 mA cm^−2^	~2000 h (symmetric Zn|Zn)	—/CE 99%/—	—	Equalized ion distribution; dendrite suppression; quasi-solid safety	[74]
Zn-ion (aqueous)	PDMC-co-NaSS	Gel electrolyte	Copolymer of cationic METAC and anionic NaSS	σ: 27.3 mS cm^−1^	5 A g^−1^	600 cycles	151.2 mAh g^−1^; CE 98–103%	—	Uniform Zn^2+^ migration; self-healing with GO/Laponite reinforcement	[75]
Zn-ion (aqueous)	PSBPP	Gel electrolyte	Zwitterionic network	—	5 A g^−1^	600 cycles	151.2 mAh g^−1^; CE ~98–103%	—	Enhanced conduction and mechanical toughness	[76]
Zn-ion (aqueous)	PSBMA (quasi-solid)	Gel electrolyte	Zwitterionic sulfobetaine + acrylamide in Zn(ClO_4_)_2_; gradient interface	σ: 6.48 mS cm^−1^	—	1000 cycles	240 mAh g^−1^; CE 99.18% (Zn|Cu over 1000 cycles)	—	Homogeneous ionic environment; suppressed parasitic reactions	[77]
Zn-ion (aqueous, antifreeze)	PSBMA hydrogel	Gel electrolyte	Water-retentive zwitterion hydrogel	σ: 1.6 mS cm^−1^ (−21 °C); 34.8 mS cm^−1^ (80 °C)	1 A g^−1^	500 cycles	154.1 mAh g^−1^	−21 °C to 80 °C	Stable low-T conduction; quasi-solid safety	[78]
Zn-ion (aqueous)	PSBMA (matrix for Zn-ion cathode)	Electrolyte environment	Homogeneous field distribution	—	—	3500 cycles	363.1 mAh g^−1^	—	Dendrite suppression; long life	[79]
Zn-ion (aqueous)	PMEAP	Gel electrolyte	Zwitterionic phosphate-bearing polyampholyte	—	1 C (cell)/5 A g^−1^	10,000 cycles (cell)	270 mAh g^−1^; CE 99%	—	Suppressed dendrite growth; stable cycling	[80]
Zn-ion (DES eutectogel)	PSPE eutectogel	Eutectogel electrolyte	DES (ChCl:EG:urea = 1:2:1) + zwitterionic monomer; water-free	σ: 7.1 mS cm^−1^ (20 °C); wide electrochem. window	1 mA cm^−2^ (symm. Zn)/1 A g^−1^ (full cell)	>2200 h (Zn|Zn); 500 cycles (V_2_O_5_)	73.9% capacity retention (500 cycles)	60 °C stability (87.9% mass retained, 55 h)	Side-reaction suppression; flexible operation	[81]
Zn-ion (coating)	P(SBMA-co-BuA)	Water-blocking anode coating	SBMA zwitterion + tert-BuA (hydrophobic)	—	1 mA cm^−2^ (symm.)/5 A g^−1^ (full cell)	2500 h (symm.)/3500 cycles (full cell)/1 A g^−1^ (hybrid SC)	Dendrite-free; HER suppressed	—	Uniform field & Zn^2+^ deposition; water exclusion	[79]
Zn-ion (coating)	PZIL (MPC in CMCS)	Protective interphase	Phosphorylcholine zwitterion; Hofmeister-enhanced contact	—	up to 40 mA cm^−2^	>1000 h (symm.)	High-rate stability; CE high (reported)	—	Chelation with Zn^2+^; side-reaction suppression	[80]
Li-ion (gel/SSE interphase)	Polyampholyte-derived AIL	Artificial interphase layer	Charge-balanced layer improving Li^+^ transport	σ: 7.5 × 10^−5^ S cm^−1^; t^+^(Li^+^) = 0.81	1 mA cm^−2^	Li plating/stripping up to 1400 h	—	—	Stabilizes LMA; low impedance	[81]
Li-ion (solid/gel electrolyte)	CBMA + flexible segment (G4)	Zwitterion-rich gel/solid electrolyte	CBMA zwitterion + tetraethylene glycol dimethyl ether	—	Symm. Li|Li at 30 °C	>5500 h; overpotential ~0.19 V	LFP: 99.9% retention (320 cycles, 30 °C); NCM811: 62.5% after 54 cycles	—	Optimized Li^+^/anion distribution; robust solid-state performance	[82]
Li-ion (dual-network PE)	Poly(zwitterion) + inorganic (TEOS)	Solid polymer electrolyte	Simultaneous zwitterion polymerization + non-hydrolytic sol–gel	σ: 0.44 mS cm^−1^ (30 °C); window > 5 V	—	—	—	—	Strength 0.75 MPa; elongation 560%; dynamic ion channels	[83]
Li-ion (ionogel)	Zwitterion-cross-linked ionogel (pCBMA dominant)	IL-based gel electrolyte	Zwitterionic units disrupt Li^+^–TFSI^−^ clusters	σ(Li^+^): 0.23 → 0.44 mS cm^−1^; t^+^: 0.23 → 0.37 (room T)	—	—	—	Low-T capable	Faster ion transport; selective Li^+^ conduction	[38]
Li–S (separator coating)	PVIPS-grafted Nafion (ZIGLN)	Functional separator (cathode side)	Imidazolium zwitterion grafted onto lithiated Nafion	σ: 1.35 × 10^−3^ S cm^−1^; t^+^: 0.75	High current densities; long-term	>1200 h (low ΔV); 500 cycles in Li–S	460 mAh g^−1^ at 1 C; shuttle suppressed	—	Dendrite inhibition; LiPS blocking; guided Li^+^ nucleation	[84]

Here, σ = ionic conductivity; t^+^ = cation transference number; CE = Coulombic efficiency. ‘—’ indicates not reported.

**Table 4:** Polyampholyte-based electrolytes for supercapacitors.

System	Polyampholyte/Zwitterion	Role in Cell	Electrodes	Medium/Formulation Highlights	Ionic Conductivity (mS cm^−1^)	Specific Capacitance (Value, Condition)	Energy Density	Power Density	Retention/Cycles	Temperature Window	Special Features/Notes	Ref.
Supercapacitor (aqueous, antifreeze)	AAm–Proline	Hydrogel electrolyte (anti-freezing)	AC	Proline-based zwitterionic hydrogel enabling sub-zero ion transport	4.2 at −40 °C	145.8 mF cm^−2^ (0.5 A g^−1^)	5.1 Wh kg^−1^	0.125 kW kg^−1^	—	Down to −40 °C	Green, flexible electrolyte; stable low-T performance	[85]
Supercapacitor (gel, redox-assisted)	PPDE (PSBMA) + EV additive	Gel electrolyte with redox additive	AC	Poly([2-(methacryloyloxy)ethyl]dimethyl-(3-sulfopropyl)ammonium hydroxide) + LiCl + ethyl viologen	20	677 F g^−1^	542 Wh kg^−1^	2.88 kW kg^−1^	65%	—	Redox-enabled faradaic boosting of capacitance	[86]
Supercapacitor (antifreeze hydrogel)	PSBMA (polySH)	Hydrogel electrolyte and strain sensor	AC	Zwitterion-rich network promoting LiCl dissociation; non-freezable water	12.6 at −40 °C	178 mF cm^−2^ (60 °C); 134 mF cm^−2^ (−30 °C)	—	—	95.5% after 30 days at −30 °C	−30 °C to 60 °C	Elastic, adhesive hydrogel; maintains capacitance at sub-zero T	[87]
Wearable/flexible SC (quasi-solid)	P(NaSS-co-DMAEA-Q-co-UM)	Polyampholyte hydrogel electrolyte	Au@PET	Random co-polymer; salt-enhanced conductivity	13.2	4.46 F g^−3^	3.84 Wh kg^−1^	196.8 W kg^−1^	87.1% after 10,000 cycles	—	Self-healing; strong interfacial linkage; low R_ct	[88]
Flexible SC (antifreeze, acid-doped)	P(NaSS-co-DMAEA-Q) + BAGU	Hydrogel electrolyte	AC	Cross-linked with BAGU; doped with H_3_PO_4_	6.88 at −30 °C; 8.23 (−30 to 20 °C)	104.9 mF cm^−2^	—	—	97.01% at 20 °C; 45.77% at −30 °C after 500 cycles	−30 °C to 20 °C	Improved anti-freezing via hydrogen-bond immobilization of free water	[89]
Quasi-solid ZIHS (adhesive)	LiA + DAC polyzwitterionic gel	Gel electrolyte (binder-like adhesion)	AC	Anionic and cationic functionalities for strong adhesion	15 at −20 °C	241 F g^−1^	34 Wh kg^−1^	598 W kg^−1^	92% after 10,000 cycles	—	Enhanced mechanical flexibility and self-healing	[90]
Supercapacitor (IL-assisted)	VIPS * + IL (SPMA) *	Hydrogel electrolyte (polyzwitterion + ionic liquid)	AC	3-(1-vinyl-3-imidazolio)propanesulfonate with SPMA; IL boosted transport	3100	108.8 F g^−1^ (1 A g^−1^)	9.67 Wh kg^−1^	0.408 kW kg^−1^	99% after 1000 cycles	—	High mobility from zwitterion–IL synergy	[91]
Stretchable micro-SC	VBIPS + IL (EMIM-BF_4_)	Double-network polyzwitterionic electrolyte	PANI-CC	Solvent-exchange IL loading; robust DN network	7.24	227.7 mF cm^−2^ (0.5 mA cm^−2^)	32 μWh cm^−2^	0.504 mW cm^−2^	79.42% (9 °C), 85.6% (−20 °C; 25 °C; 50 °C)	−60 °C to 50 °C	Strong tensile strength (1.9 MPa); outstanding rate capability	[92]
All-hydrogel integrated SC	P(NaSS-co-DMAEA-Q)-ACP	Electrolyte + electrode (self-adhesive)	P(NaSS-co-DMAEA-Q)-ACP + AC	Electrostatic self-adhesion; energy-dissipative network	—	128.9 mF cm^−2^ (1 mV s^−1^)	5.6 μWh cm^−2^	0.1 mW cm^−2^	90%	—	Compressible, soft device for wearables	[93]
IL-electrolyte SC	PYR_14_TFSI *	Ionic liquid electrolyte (benchmark)	AC	Room-temperature IL providing wide window	60	60 F g^−1^ (20 mV s^−1^)	31 Wh kg^−1^	8.6 kW kg^−1^	—	—	Reference IL system for comparison	[94]
IL-based composite SC	BMIM-PF_6_ (plasma-treated composite)	IL composite electrolyte	AC	Plasma-treated composite with IL for improved wetting	—	24.8 F g^−1^; 357 F g^−1^	—	—/458 W kg^−1^	—	—	Enhanced flexibility and performance	[95,96]
IL/solvent hybrid SC	P/AC/IBOB in ethanol and IL	Ionogel-like electrolyte	AC	Lithium oxalate borate salt in PC; IL hybridization	—	40 F g^−1^ (2 A g^−1^); 89 F g^−1^ (2 A g^−1^)	50 Wh kg^−1^; 60 Wh kg^−1^	—/763 W kg^−1^	—	—	Improved energy density via IL hybrid	[97,98]
Salt-in-IL hydrogel SC	ZrSO_4_ in EG; CaCl_2_ in H_2_O; ZnSO_4_ in H_2_O	Hydrated ionogel electrolytes	AC	Hydrogen-bond network electrolytes for cold-resistance	6.09 (−1 °C); 11.01 (−50 °C); 40.6 mS cm^−1^	121 F g^−1^ (0.1 A g^−1^); 60 F g^−1^ (2.0 A g^−1^); 436 F g^−1^	17.2 Wh kg^−1^; 13.48 Wh kg^−1^	1.12 kW kg^−1^; 11.84 kW kg^−1^	92.1% (various systems); 100% (ZnSO_4_/H_2_O)	Down to −50 °C (CaCl_2_/H_2_O)	Cold-resistant ion transport with robust networks	[99,100,101]

Here, AC, activated carbon; IL, ionic liquid; DN, double network; ZIHS, zinc-ion hybrid supercapacitor; VIPS, 3-(1-vinyl-3-imidazolio)propanesulfonate; SPMA, 3-sulfopropyl methacrylate; EMIM-BF_4_, 1-ethyl-3-methylimidazolium tetrafluoroborate; PYR_14_TFSI, N-butyl-N-methylpyrrolidinium bis(trifluoromethanesulfonyl)imide; BMIM-PF_6_, 1-butyl-3-methylimidazolium hexafluorophosphate; PANI-CC, polyaniline-carbon cloth; EV, ethyl viologen; PC, propylene carbonate; IBOB, lithium bis(oxalato)borate; EG, ethylene glycol; R_ct, charge-transfer resistance.

## Data Availability

No new data were created or analyzed in this study. Data sharing is not applicable to this article.
